# Metabolic syndrome and cognition: A systematic review across cognitive domains and a bibliometric analysis

**DOI:** 10.3389/fpsyg.2022.981379

**Published:** 2022-11-09

**Authors:** Myrto Koutsonida, Georgios Markozannes, Emmanouil Bouras, Eleni Aretouli, Konstantinos K. Tsilidis

**Affiliations:** ^1^Department of Hygiene and Epidemiology, School of Medicine, University of Ioannina, Ioannina, Greece; ^2^Laboratory of Hygiene, Social & Preventive Medicine and Medical Statistics, Department of Medicine, School of Health Sciences, Aristotle University of Thessaloniki, Thessaloniki, Greece; ^3^Department of Psychology, School of Social Sciences, University of Ioannina, Ioannina, Greece; ^4^Laboratory of Cognitive Neuroscience, School of Psychology, Aristotle University of Thessaloniki, Thessaloniki, Greece; ^5^Department of Epidemiology and Biostatistics, School of Public Health, Imperial College London, London, United Kingdom

**Keywords:** metabolic syndrome, cognitive domains, systematic review, longitudinal design, bibliometric analysis

## Abstract

The aim of this review is to investigate the association between metabolic syndrome (MetS) and cognitive decline in distinct cognitive domains, and to perform a complementary study description through the bibliometric analysis. PubMed and Scopus databases were searched from inception to 15 December 2021 to identify longitudinal studies that examined the association of MetS with incident decline, in order to prevent reverse causality. The Preferred Reporting Items for Systematic Review and Meta-Analysis checklist was used to conduct the present systematic review. Thirty studies were included and results were analyzed across the cognitive domains of global cognition, memory, executive functions, attention, visuoconstructive abilities, and language. The majority of the studies reviewed did not report statistically significant results for most cognitive domains investigated, and decline in specific cognitive domains was not consistently associated with the presence of MetS. Meta-analyses were not conducted due to the high degree of between-study heterogeneity regarding the MetS definitions, the cognitive domains examined, the specific tests used for each cognitive domain and the different measures of association used. Bibliometric analysis revealed that most studies are conducted by research teams from USA and China, and that cognitive tasks that reflect real-life abilities are rarely examined. Future studies should employ larger sample sizes, longer follow-up periods, a global consensus for MetS definition and standardized tests of the above mentioned cognitive domains as well as problem-solving tasks with high sensitivity and specificity to clarify the impact of MetS on cognition and its underlying mechanisms.

## Introduction

Metabolic syndrome (MetS) was first described as “Syndrome X” by G. M. Reaven, as a cluster of interconnected metabolic factors that seemed to increase the risk of coronary artery disease (Reaven, 1988). Reaven suggested that resistance to insulin-stimulated glucose uptake, glucose intolerance, hyperinsulinemia, increased very-low-density lipoprotein triglyceride (LDL), decreased high-density lipoprotein cholesterol (HDL-C), and hypertension, even if not all present, could play an important role in the pathogenesis of coronary artery disease.

Since then, many prominent health organizations recommended different criteria for the definition of MetS. Despite their similarities with regards to the basic aspects of obesity, dyslipidemia, hypertension and insulin resistance, the different criteria often describe distinct clinical profiles. Table 1 summarizes the most widely used criteria from three health organizations, World Health Organization (WHO) (World Health Organization [WHO], 1999), National Cholesterol Education Program’s Adult Treatment Panel III (NCEP-ATP III) (Expert Panel on Detection, Evaluation and Treatment of High Blood Cholesterol in Adults, 2001) and International Diabetes Federation (IDF) (Alberti et al., 2005).

**TABLE 1:** Criteria used for the definition of metabolic syndrome according to WHO, NCEP-ATP III, and IDF.

	World Health Organization [WHO] (1999)	Expert Panel on Detection, Evaluation and Treatment of High Blood Cholesterol in Adults (2001)	International Diabetes Federation [IDF] (2005)
	Diabetes + ≥ 2 of the following	≥3 of the following	BMI/Central obesity + ≥ 2 of the following
Diabetes	Diabetes Mellitus OR impaired glucose tolerance OR insulin resistance		Type 2 Diabetes
Blood pressure	SBP ≥ 140 mmHg OR DBP ≥ 90 mmHg	SBP ≥ 130 mmHg OR DBP ≥ 85 mmHg	SBP ≥ 130 mmHg OR DBP ≥ 85 mmHg OR Antihypertensive medication
Fasting glucose		≥110 mg/dL	≥ 100 mg/dL
Body mass index (BMI)	> 30 kg/m^2^		>30 kg/m^2^
Central obesity	WHR > 0.9 (men) WHR > 0.85 (women)	WC > 102 cm (men) WC > 88 cm (women)	WC (ethnicity-specific values)
Triglycerides	≥150 mg/dL	≥150 mg/dL	≥ 150 mg/dL OR Lipid abnormality treatment
HDL-C	<35 mg/dL (men) < 39 mg/dL (women)	< 40 mg/dL (men) <50 mg/dL (women)	<40 mg/dL (men) <50 mg/dL (women) OR Lipid abnormality treatment
Albumin	UAE ≥ 20 μg/min ACR ≥ 30 mg/g		

SBP, systolic blood pressure; DBP, diastolic blood pressure; WHR, waist-to-hip ratio; WC, waist circumference; UAE, urinary albumin excretion rate; ACR, albumin creatinine ratio.

The prevalence estimates for MetS vary based on the criteria used for its definition, with several studies in different ethnicities reporting a difference in prevalence of 10–20% (Qiao, 2006; Churilla et al., 2007; Li et al., 2018). However, it is estimated that the global prevalence of MetS is around 25% (International Diabetes Federation [IDF], 2005).

The exact mechanisms that underlie the pathophysiology of MetS remain unclear but it is proposed that insulin resistance, oxidative stress and chronic inflammation are highly involved. Insulin resistance refers to the state of reduced cell responsiveness to normal insulin levels and it mainly occurs from an excess in free fatty acids (FFA). As a result, since insulin does not stimulate glucose transporter type 4 (GLUT4) to transfer glucose inside the cells, hyperglycemia is induced, leading to type 2 diabetes. Oxidative stress is defined as an imbalance in the production of reactive oxygen species (ROS), over the capability of the cell to have an effective antioxidant response. ROS can be overproduced by mitochondria during consumption of a high-energy meal, and are associated with increased LDL and decreased HDL-C levels. Regarding chronic inflammation, one possible mechanism is via the adipocytes within the adipose tissue. Accumulation of body fat is induced in response to an excess nutritional energy intake that overcomes metabolic requirements at obesity. Adipocytes release pro-inflammatory cytokines, such as tumor necrosis factor-alpha (TNF-α) and interleukin-6 (IL-6), which in turn contribute to the development of hypertension and insulin resistance (McCracken et al., 2018; Xu et al., 2018).

All the above mentioned pathogenetic mechanisms and all the components of MetS have been individually linked with cognitive impairment. Insulin resistance (Ma et al., 2015; Ekblad et al., 2017), oxidative stress (Hajjar et al., 2018), and chronic inflammation (Sartori et al., 2012) are conditions that commonly coexist with lower cognitive abilities. Diabetes (Callisaya et al., 2019; Zhang et al., 2019), hypertension (Ihle et al., 2018; Forte et al., 2019), obesity (Fellows and Schmitter-Edgecombe, 2018; Yang et al., 2018), and dyslipidemia (Ma et al., 2017; Fu et al., 2021) have also been correlated with decline in memory, executive functions and global cognition.

Considering the high prevalence of neurocognitive disorders (Alexander et al., 2015), delaying the onset and progression of these disorders by various means, such as appropriate cognitive training programs is critical (Hill et al., 2017). However, for successful cognitive training programs to be designed, there needs to be previous knowledge about the cognitive domains affected. Although some recent reviews have examined the association of MetS with cognition, there is still no consensus about their relationship, partly due to inconsistency in the cognitive domains and tests used for assessment. Five systematic reviews (Hao et al., 2011; Crichton et al., 2012; Yates et al., 2012; Farruggia and Small, 2019; Tahmi et al., 2021) and two meta-analyses (Siervo et al., 2014; González-Castañeda et al., 2021) have reported small but negative association of MetS with cognition, while two other reviews have shown inconclusive results (Assuncao et al., 2018; Alcorn et al., 2019). Of these studies, only two analyzed the available data for the association of MetS and cognitive performance across different cognitive domains but they either included only cross-sectional studies (Alcorn et al., 2019) or a mix of different study designs (González-Castañeda et al., 2021), complicating the temporal sequence and the establishment of causality. The scope of the present review is twofold. First, we aimed to investigate the association between MetS and incident decline in distinct cognitive abilities including only prospective studies which are less prone to biases such as reverse causation. Second, we aimed to perform a complementary study description analysis and present the general trends and research gaps in the field of cognition in individuals with MetS through a bibliometric analysis.

## Methods

### Search strategy and study selection

PubMed and Scopus databases were searched from inception to 15 December 2021 using the following search terms: (metabolic syndrome) AND (cognition OR cognitive impairment OR cognitive decline OR cognitive function OR cognitive dysfunction). Further search terms for the specific cognitive domains (”Memory,” “Working memory,” “Short-term memory,” “attention,” “Language,” “Verbal fluency,” “Executive functions,” “Construction,” “Visuoconstruction,” “Mental functions”) were added to the algorithm as a sensitivity analysis but they did not provide any additional eligible articles. Screening of the retrieved articles was accomplished by two researchers (MK and GM). Discrepancies were adjudicated by an expert epidemiologist (KKT).

The retrieved articles were screened based on the following inclusion criteria: (a) human studies in adults (18 years or older), (b) with no serious comorbid psychiatric or neurological disease, (c) referring to MetS as a combined factor and not its individual components, (d) the outcome of interest was cognitive abilities examined using neuropsychological tests, (e) studies utilizing a prospective design. Studies on related cognitive syndromes such as mild cognitive impairment or dementia were excluded. We included studies irrespective of language of publication, type of diagnostic criteria used for the definition of MetS, and specific neuropsychological tests used (ex. domain-specific or global cognition).

**FIGURE 1:**
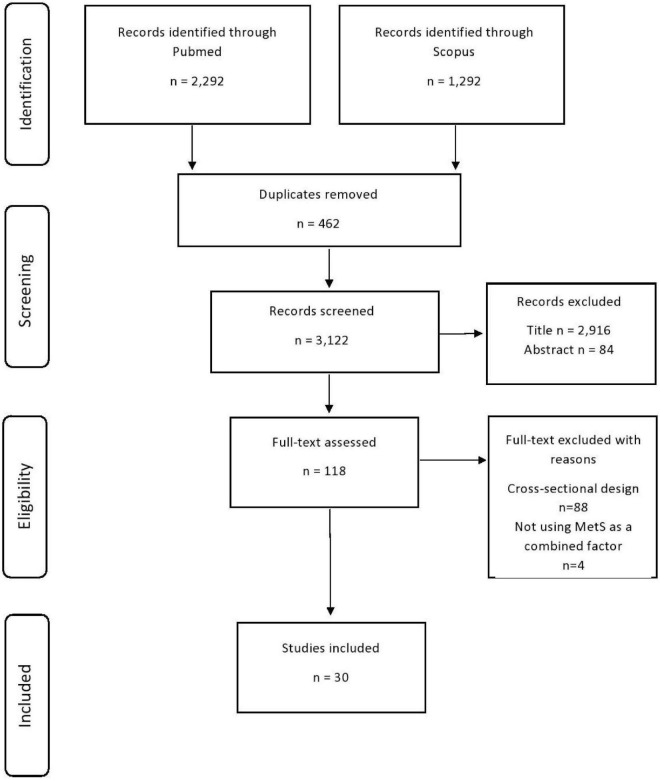
Flowchart of study selection.

References from review articles (Hao et al., 2011; Crichton et al., 2012; Yates et al., 2012; Siervo et al., 2014; Assuncao et al., 2018; Alcorn et al., 2019; Farruggia and Small, 2019; González-Castañeda et al., 2021; Tahmi et al., 2021) were screened for potentially missed papers. The titles of articles were screened first and then abstract and full-text review was performed if articles were deemed relevant at each review phase. Figure 1 presents the flowchart of study selection according to the Preferred Reporting Items for Systematic Reviews and Meta-Analyses (PRISMA) guidelines (Page et al., 2021).

### Data extraction and quality assessment

Data concerning article characteristics (i.e., title, lead author, publication year), participants’ characteristics (country of origin, age and gender distributions, recruitment, baseline and follow-up period, sample size of MetS and control group), definition of MetS (criteria, time of assessment, duration of diagnosis), outcomes (neuropsychological tests, cognitive domain, metric for test result) and methodology (statistical analysis, effect estimate and measure of variability, covariates) were extracted by one researcher (MK) and confirmed by a second researcher (GM). The cognitive tests used in each study were classified into cognitive domains according to the classification by Lezak et al. (2004) in global cognition, memory, executive functioning, attention, language and visuoconstructive abilities.

Quality of each included study was assessed by the Newcastle–Ottawa Scale (NOS) for cohort studies (Wells et al., 2014). NOS is a 9-point scale that assesses study quality in three domains, sample selection, comparability of sample, and outcome assessment. The NOS criteria were used to qualitatively assess risk of bias regarding generalizability and selection bias, exposure and outcome measurement error, reverse causation bias, residual confounding, adequacy of follow-up and bias due to loss to follow-up.

### Statistical analysis

We did not perform a meta-analysis of the included studies as originally intended, due to substantial heterogeneity among included studies concerning the cognitive domains examined, the specific tests used, the types of final score used and the reported measures. For instance, the outcome was analyzed either as continuous or dichotomous and either as the final score at follow-up period (in a raw or in a transformed form, i.e., *z*-scores) or as the change in score (i.e., difference between baseline and follow-up score, score decline based on predefined normative data). Attempts to overcome these limitations usually include approaches such as computing crude mean differences utilizing the raw data or computing a harmonized effect size across studies (i.e., standardized mean difference). However, computing crude estimates will not account for any confounder, leading to serious risk of bias, and computing the standardized mean difference is based on the assumption that the data rise from an independent samples *t*-test which will lead to largely biased estimates when it is implemented in estimates from more complex statistical models, such as multiple linear regression. Therefore, a qualitative synthesis was considered the more appropriate way for presenting the results.

We used albatross plots (Harrison et al., 2017) in order to depict the results of each study according to its sample size and identify potential sources of heterogeneity. The albatross plots are scatter plots of study sample sizes against two-sided *p*-values separated by the observed direction of effect, and they illustrate the presence of small study bias.

### Bibliometric analysis

A bibliometric analysis was conducted to quantitatively analyze and describe the trends in the characteristics of the included studies over the years. Specifically, the analyses included an overview of the trend topics, the top cited studies, the most productive countries and the most relevant keywords. The analysis was performed using the Bibliometrix package in R v 3.5.3.

## Results

### Results of the search

A total of 3,584 records were identified from the initial search (Figure 1). Of those, 462 duplicate records were removed, 3,000 records were excluded based on the title and abstract, and 92 based on full-text. The reasons for excluding full-text records were not a prospective cohort design (*n* = 88) and not using MetS as a combined factor (*n* = 4). Ultimately, 30 relevant prospective studies included in the qualitative synthesis (Yaffe et al., 2004, 2007; Xiong et al., 2006; Komulainen et al., 2007; van den Berg et al., 2007; Ho et al., 2008; Knopman et al., 2009; Yaffe et al., 2009; Akbaraly et al., 2010; Lee et al., 2010; Raffaitin et al., 2011; Creavin et al., 2012; Katsumata et al., 2012; McEvoy et al., 2012; Liu et al., 2013; Watts et al., 2013; Dearborn et al., 2014; Harrison et al., 2015; Gallagher et al., 2016; Viscogliosi et al., 2016, 2017; Neergaard et al., 2017; Overman et al., 2017; Shigaeff et al., 2017; Bangen et al., 2019; Kazlauskaite et al., 2020; Przybycien-Gaweda et al., 2020; Soldevila-Domenech et al., 2021; Wu and Chen, 2021; Wang et al., 2021).

### Characteristics of included studies

The 30 included studies comprised of 62,471 participants (54.9% women) with age at baseline cognitive assessment over 50 years old. Twelve studies were conducted in Europe, nine in USA, seven in Asia, one in Brazil and one study was multinational. Four studies included only women and five studies included only men. The sample size ranged from 43 to 7,035 participants (median sample size 1,178 participants) with 16 studies including more than 1,000 participants. The follow-up period ranged from 1 to 16 years (median duration 5 years). Most studies (*n* = 21, 70%) used the NCEP-ATP III criteria for diagnosis of MetS. The most commonly used neuropsychological test was the Mini-Mental State Examination (MMSE) (Folstein et al., 1975), either alone (in six studies) or in combination with other neuropsychological tests that assess memory, attention and executive functions (in eight studies) (Table 2).

**TABLE 2:** Basic details of the studies included in the systematic review.

Lead author (Year)	Participant source (Country)	Sample size, n	Baseline female, %	Baseline mean age, years	Follow-up, years^a^	MetS criteria	MetS diagnosis, %	Cognitive domains	Neuropsychological test
Akbaraly et al., 2010	Whitehall II study (UK)	4,150	26.1^b^	61.1	10	NCEP-ATP III	8.3	G, M, Ef, L	20-word free recall test, A H4-1, Mill Hill Vocabulary test, VF, MMSE
Bangen et al., 2019	Framingham Heart Study, Offspring Cohort (USA)	2,892	53.7	62.3	15	NCEP-ATP III	70	G, M, Ef	Logical Memory (WMS), Visual Reproduction (WMS), Verbal Paired Associates (WMS), TMT-A, TMT-B, Digit Span (WMS), Similarities (WAIS), FAS, CF (Animal), Hooper Visual Organization Test, 30-item Boston Naming Test
Creavin et al., 2012	Caerphilly Prospective study (UK)	1,225	0	52.8^c^	13	WHO	10	G, Ef, A	CAMCOG, AH4-1, four choice reaction time
Dearborn et al., 2014	Atherosclerosis Risk in Communities cohort (USA)	10,478	56.2	54.1	9	AHA	36.5	M, Ef, A	Delayed word recall, DSST (WAIS-R), FAS
Gallagher et al., 2016	English Longitudinal Study of Aging (UK)	5,590	55.1	67.2	4	IDF	26.7	M, Ef	Word lists developed for the Health and Retirement Study, CF (Animal)
Harrison et al., 2015	Newcastle 85 + Study (UK)	406	62.2	85	5	NCEP-ATP III^d^	27.4	G, M, A	MMSE, Simple Reaction Time (CDR), Choice Reaction Time (CDR), Digit Vigilance Task (CDR), word recognition task
Ho et al., 2008	Singapore Longitudinal Aging Studies (Singapore)	1,352	66.3	65.4	2	IDF	26.3	G	Chinese MMSE
Katsumata et al., 2012	Keys to Optimal Cognitive Aging Project (Japan)	148	74.3	85	3	NCEP-ATP III	33.1	G, M, Ef	Japanese MMSE, Scenery Picture Memory Test, VF (initial letter ka)
Kazlauskaite et al., 2020	Study of Women’s Health Across the Nation inception cohort (USA)	2,149	100	46.4	13	Cardio-Metabolic Health Alliance	29.5	M, Ef, A	SDMT, East Boston memory test, Digit Span Backward (WAIS-IV)
Knopman et al., 2009	Atherosclerosis Risk in Communities cohort (USA)	1,130	62	59	14	NCEP-ATP III	46	M, Ef, A	Delayed word recall, DSST (WAIS-R), FAS
Komulainen et al., 2007	North Karelia Project (Finland)	101	100	63.7	12	NCEP-ATP III	13	G, M, A	MMSE, Word Recall Test, prospective memory task, Stroop Test, Letter-Digit Substitution Test
Lee et al., 2010	Gwangju Dementia and Mild Cognitive Impairment Study (Korea)	596	58.4	72	2	NCEP-ATP III	34.7	G	Korean MMSE
Liu et al., 2013	Longitudinal Older Veteran study (Taiwan)	229	0	82.4	1	NCEP-ATP III	22.5	G	MMSE
McEvoy et al., 2012	Rancho Bernardo Study (USA)	993	58.8	66.8	16	NCEP-ATP III	11	G, M, Ef	MMSE, TMT-B, Buschke-Fuld Selective Reminding Test, Verbal Category Fluency
Neergaard et al., 2017	Prospective Epidemiological Risk Factor Study (Denmark)	1,759	100	68	15	IDF		G, Ef	Short Blessed Test, CF (Animal)
Overman et al., 2017	European Male Aging Study (Belgium, UK, Italy, Poland, Sweden, Spain, Hungary, Estonia)	1,965	0	59.3	5	NCEP-ATP III^d^	28.8	M, A, V	ROCF, DSST (WAIS-III), Camden Topographical Recognition Memory
Przybycien-Gaweda et al., 2020	Singapore Longitudinal Aging Studies (Singapore)	262	62.6	65.3	5	NCEP-ATP III^e^	27	G, M, Ef, A	SDMT, Design Fluency, Block Design, TMT-A, TMT-B, Digit Span, CF (Animal), Visual Reproduction, RAVLT
Raffaitin et al., 2011	Three-City Study (France)	7,035	61	73.4	4	NCEP-ATP III^d^	15.8	G, M, Ef	MMSE, Isaacs Set Test, BVRT
Shigaeff et al., 2017	Universidade Federal de São Paulo, Serviço Social do Comércio (Brazil)	43			1	NCEP-ATP III^d^	50	M, Ef, A, V, L	TMT -A, TMT -, Digit Span (WAIS-III), RAVLT, WCST, Cubes (WAIS- III), Boston Naming Test, FAS
Soldevila-Domenech et al., 2021	PREDIMED-Plus-*Cognition* sub-study (Spain)	487	50.5	65.2	3	IDF	100	G, M, Ef	RAVLT, ROCF, SDMT, Stroop Test, Iowa Gambling Task, Conners’ Continuous Auditory Test of Attention
van den Berg et al., 2007	Leiden 85-plus Study (Netherlands)	497	63	85	5	NCEP-ATP III	42	G, M, Ef, A	MMSE, Stroop Test, Letter Digit Coding Test, 12 Word Learning Test
Viscogliosi et al., 2016	“Merry House” clinic (Italy)	104	56	80.2	1	NCEP-ATP III	29.8	Ef	CDT
Viscogliosi et al., 2017	Finland, Italy, and the Netherlands Elderly Study (Italy)	195	0	76.1	10	NCEP-ATP III	24.6	G	MMSE
Wang et al., 2021	Department of Geriatric Medicine at Xuanwu Hospital (China)	202	34.2	75.9	1	Chinese Medical Association	49.5	G	MMSE
Watts et al., 2013	Brain Aging Project (USA)	66	58	74.2	2	Factor analysis	33	G, M, A	MMSE, Logical Memory (WMS-R), Free and Cued Selective Reminding Task (WMS-R), Letter–number sequencing (WAIS), DSST (WAIS), Stroop Test
Wu and Chen, 2021	Taiwan biobank (Taiwan)	5,693	60.1	63.7	10	NCEP-ATP III	27.5	M, A, V, L	MMSE
Xiong et al., 2006	Duke Twins Study of Memory in Aging (USA)	3,573	0	65.8	12	WHO	4.5	G	TICS-m
Yaffe et al., 2004	Health, Aging and Body Composition study (USA)	2,632	52	73.6	5	NCEP-ATP III	38.6	G	3MS
Yaffe et al., 2007	Sacramento Area Latino Study on Aging (USA)	1,624	58.5	70.5	3	NCEP-ATP III	44	G, M	3MS, Delayed Word-List Recall
Yaffe et al., 2009	Multiple Outcomes of Raloxifene Evaluation trial (25 countries, mainly USA and Europe)	4,895	100	66.2	4	NCEP-ATP III	10.2	G	Short Blessed Test

NCEP-ATP III, National Cholesterol Education Program’s Adult Treatment Panel III; WHO, World Health Organization; AHA, American Heart Association; IDF, International Diabetes Federation; G, global cognition; M, memory; Ef, executive functions; L, language; A, attention; V, visuoconstructive abilities; AH4-1, Alice Heim 4 test-part 1; VF, Verbal Fluency test; MMSE, Mini-Mental State Examination; WMS, Wechsler Memory Scale; TMT-A, Trail Making Test-part A; TMT-B, Trail Making Test-part B; FAS, phonemic verbal fluency test with the letters F, A, and S; CF, Category Fluency test; CAMCOG, Cambridge Cognition Examination; DSST, Digit Symbol Substitution Test; WAIS-R, Wechsler Adult Intelligence Scale-Revised; CDR, Cognitive Drug Research; SDMT, Symbol Digit Modalities Test; ROCF, Rey–Osterrieth complex figure; RAVLT, Rey Auditory Verbal Learning Test; BVRT, Benton Visual Retention Test; WCST, Wisconsin Card Sorting Test; CDT, Clock Drawing Test; TICS-m, Modified Telephone Interview for Cognitive Status; 3MS, Modified Mini-Mental State Examination.

^a^For cognition measure.

^b^Not baseline.

^c^Median.

^d^Revised 2005.

^e^Revised 2009.

### Quality assessment

Quality assessment for each study is shown in Supplementary Table 1. Most studies employed a representative sample of the general population (*n* = 25, 83.3%) with minimal influence of selection bias (*n* = 29, 96.7%). Presence of MetS was ascertained using anthropometric, clinical and biochemical measurements (*n* = 29, 96.7%) and almost all studies administered a validated neuropsychological test to ascertain cognition (*n* = 28, 93.3%). Almost half of the studies controlled for important confounders, namely gender, age and education (*n* = 14, 46.7%), whereas fewer studies controlled also for additional factors, such as smoking, alcohol use, physical activity and depression (*n* = 11, 36.7%); five studies (16.7%) did not adjust for all the important confounders (gender, age, education). Even though a large number of included studies had an adequate follow-up period of 4 years or above (*n* = 21, 70%), losses to follow-up was a common limitation (*n* = 15, 50%). Another limitation was reverse causation bias, as a considerable number of included studies did not check for presence of cognitive decline at baseline (*n* = 13, 43.3%).

### Metabolic syndrome and cognitive abilities across domains

Over half of the studies (*n* = 17, 57%) concluded that MetS may lead to faster rates of cognitive decline in at least one cognitive domain. Regarding the specific cognitive domains, 22 studies investigated the association of MetS with global cognition, 19 with memory, 16 with executive functions and 12 with attention. Three studies investigated the association of MetS with visuoconstructive abilities and another 3 studies with language (Table 3).

**TABLE 3:** Summary of the results by cognitive domain, effect metric and adjustments of the included studies.

	Global				Memory				Executive functions				Attention				
Study	Effect metric	Baseline levels	Final levels	Change in levels	Effect metric	Baseline levels	Final levels	Change in levels	Effect metric	Baseline levels	Final levels	Change in levels	Effect metric	Baseline levels	Final levels	Change in levels	Adjustments
Akbaraly et al., 2010	F		NS		F		NS		F		NS						Age, gender, education or occupation, marital status, smoking, alcohol, physical activity, depression, coronary heart disease
Bangen et al., 2019	β	–	–	NS	β	–	NS	NS	β	–	–	NS					Age, gender, education, smoking
Creavin et al., 2012	β		NS						β		–		β		NS		Age, social class, smoking, alcohol, premorbid cognition
Dearborn et al., 2014, women					β	–		NS	β	–		NS	β	NS		NS	Age, education, race-center, smoking, alcohol, history of coronary heart disease
Dearborn et al., 2014, men					β	–		NS	β	–		NS	β	NS		NS	
Gallagher et al., 2016					β			NS	β			NS					Age, gender, duration of follow-up, cognitive status at baseline
Harrison et al., 2015	β	NS		NS	β	NS		NS					β	NS		NS	Gender, education, smoking, alcohol, marital status, history of stroke/ischemic heart disease/heart failure
Ho et al., 2008	OR	–		–													Age, gender, education, smoking, alcohol, depression, level of leisure activities, APOE-e4, baseline MMSE, years of follow-up
Katsumata et al., 2012	β	NS		NS	β	NS		NS	β	–		NS					Age, gender, education
Kazlauskaite et al., 2020					β	NS NS		NS NS	β	NS		NS	β	NS		–	Age, education, financial strain, site–race, smoking, alcohol, physical activity, sleep difficulty, depression, anxiety, vasomotor symptoms, menopausal stages/hormone therapy
Knopman et al., 2009					β			NS	β			–	β			NS	Age, gender, education, race
Komulainen et al., 2007	OR	NS	NS		OR		–						OR		NS		Age, education, depression
Lee et al., 2010	F	–		–													Age, gender, education, height, weight, baseline MMSE, years of follow-up
Liu et al., 2013	OR	NS		+													Smoking
McEvoy et al., 2012, women	β	NS		NS	β	NS		NS	β β	NS NS		– NS					Age, education, depression, practice effect
McEvoy et al., 2012, men	β	NS		NS	β	NS		NS	β β	NS NS		NS NS					
Neergaard et al., 2017	OR OR		NS NS						OR		NS						Age, education, smoking, alcohol, physical activity, hormone replacement therapy
Overman et al., 2017					β, OR β, OR			NS NS					β, OR			NS	Age, education, center, smoking, alcohol, depression, physical activity, heart disease/stroke
Przybycien-Gaweda et al., 2020	β	NS	–	NS	β	–	–	NS	β	–	–	NS	β β	– NS	– –	NS NS	Age, gender, education, smoking, alcohol, depression, leisure time activity, APOE-e4, cardiovascular disease/stroke
Raffaitin et al., 2011	HR	–		–	HR	–		–	HR	–		NS					Age, gender, education, city center, smoking, depression, APOE-e4, cardiovascular disease, score on test at baseline
Shigaeff et al., 2017					U	NS NS NS	NS NS NS		U	NS NS NS NS	NS NS NS –		U	NS NS	NS NS		Age, education
Soldevila-Domenech et al., 2021	β			NS	β			NS	β			NS					Age, gender, education, IQ, smoking, baseline weight, prediabetes, diabetes, use of treatment for high cholesterol, use of tranquilizers or sedatives, intervention group
van den Berg et al., 2007	β	NS		+	β	NS NS		NS NS	β	NS		+	β	NS		+	Gender, education, depression
Viscogliosi et al., 2016									*std*. β	–		–					Potentially confounding variables
Viscogliosi et al., 2017	*std*. β	NS		–													Age, education, marital status, depression, APOE-e4, cerebrovascular disease, baseline MMSE, incidence of ADLs disability, interaction term (MetS)*(baseline MMSE score)
Wang et al., 2021	F	–	–														Age, gender, education, professional characteristics, smoking, alcohol, living status
Watts et al., 2013	*std*. β	NS	NS	NS	*std*. β	NS	NS	NS					*std*. β	NS	NS	NS	Age, gender, education, APOE-e4
Wu and Chen, 2021		–			HR, KM		NS	NS					HR, KM		– NS NS	– NS NS	Age, gender, education, smoking, coronary artery disease, hypertension, diabetes type 2
Xiong et al., 2006	F			NS													Age at Time 3, education, smoking, alcohol, baseline TICS-m
Yaffe et al., 2004	RR	NS	–														Age, education, race, alcohol, depression, stroke, statin use, baseline 3MS
Yaffe et al., 2007	β	NS		–	β	NS		NS									Age, gender, education, born in the USA, smoking, alcohol, depression, history of stroke, history of myocardial infarction, missing pattern indicator
Yaffe et al., 2009	OR		NS														Age, education, race, depression, raloxifene hydrochloride treatment

A plus (+) sign indicates a higher performance of participants with metabolic syndrome and a minus (–) sign a lower performance in the respective test. F, *F*-test (ANOVA); U, Mann–Whitney test; β, beta coefficient; *std*. β, standardized beta coefficient; OR, odds ratio; HR, hazard ratio; RR, risk ratio; KM, Kaplan–Meier curves; NS, not significant; MetS, metabolic syndrome; APOE-e4, e4 allele of the apolipoprotein E; MMSE, Mini-Mental State Examination; IQ, intelligence quotient; ADLs, Activities of Daily Living; TICS-m, Modified Telephone Interview for Cognitive Status; 3MS, Modified Mini-Mental State Examination; USA, United States of America.

#### Global cognition

Global cognition refers to the level of general cognitive function of a person. It was assessed in 22 out of 30 studies with five different neuropsychological procedures.

Thirteen studies examined global cognition using the MMSE, a 30-item brief screening scale that assesses orientation, attention, memory, visuoconstructive abilities, and language. Four reported higher rates of cognitive decline in participants with MetS (Ho et al., 2008; Lee et al., 2010; Raffaitin et al., 2011; Viscogliosi et al., 2017) while another three reported no significant association (Katsumata et al., 2012; McEvoy et al., 2012; Harrison et al., 2015). One study found an association of MetS and lower cognitive levels (Wang et al., 2021) but another three found no association (Komulainen et al., 2007; Akbaraly et al., 2010; Watts et al., 2013). The studies that presented significant association of MetS with decline in cognition had larger sample size (range 195–7,035 participants, median sample size 596 participants), defined MetS with various criteria (NCEP-ATP III, IDF, Chinese Medical Association) but had shorter follow-up periods (range 1–10 years, median 2 years) compared to studies that reported null associations (range 66–4,150 participants, median 277 participants, NCEP-ATP III criteria for MetS definition in all but one study, range of follow-up period 2–16 years, median 7.5 years).

Only two studies in elderly populations aged over 75 years (Liu et al., 2013) and over 85 years (van den Berg et al., 2007) found a protective role of MetS (NCEP-ATP III criteria) on rates of cognitive decline measure by the MMSE. Authors attributed this finding to survival bias, implying that adults surviving to this age with the presence of MetS may be less susceptible to its adverse effects.

Two studies conducted in USA (Yaffe et al., 2004, 2007) utilized the Modified MMSE (3MS) (Teng and Chui, 1987) which includes four extra questions. Both studies (mean age 74 and 70 years) found that MetS (NCEP-ATP III criteria) contributed in 3- and 5-year impairment in cognitive levels and in 3-year cognitive decline, especially in elders with MetS and high inflammation markers.

Parallel forms of the MMSE were used also in four other studies that suggested no association of MetS diagnosis and cognitive levels. Namely, Yaffe et al. (2009) and Neergaard et al. (2017) examined the global cognition of 4,895 and 1,759 older women (mean age 66.2 and 66.9 years) with the Short Blessed Test (SBT) (Katzman et al., 1983), and Creavin et al. (2012) examined 1,225 older men (median age 52.8 years) with the Cambridge Cognitive Examination (CAMCOG) (Roth et al., 1986). Xiong et al. (2006) examined twin male veterans (mean age 65.8 years) with the modified Telephone Interview for Cognitive Status (TICS-m) (Welsh et al., 1993) and did not find greater decline in twins with MetS than their co-twins without MetS (WHO criteria).

Finally, some researchers evaluated global cognition using the rate of change in a composite score derived from domain-specific scores but found no evidence of association (Bangen et al., 2019; Przybycien-Gaweda et al., 2020; Soldevila-Domenech et al., 2021). Bangen et al. (2019) did find lower levels of cognitive performance at follow-up but participants with MetS had also lower levels of cognitive performance at baseline.

**FIGURE 2:**
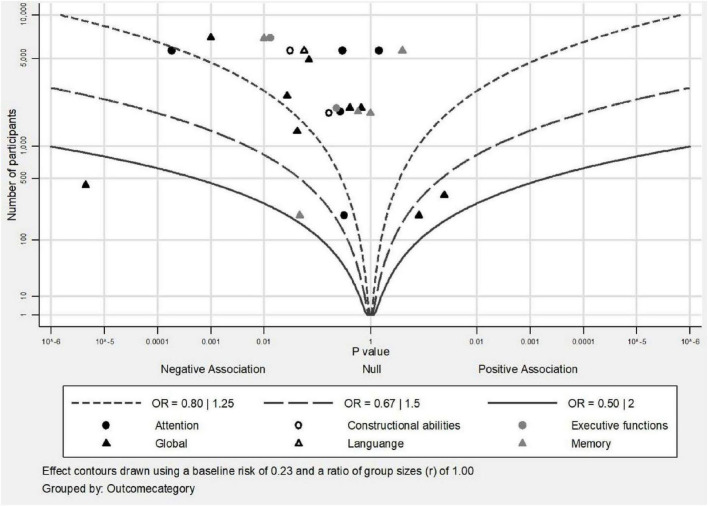
Albatross plot of studies that investigated the association of MetS with categorical outcomes of cognition.

**FIGURE 3:**
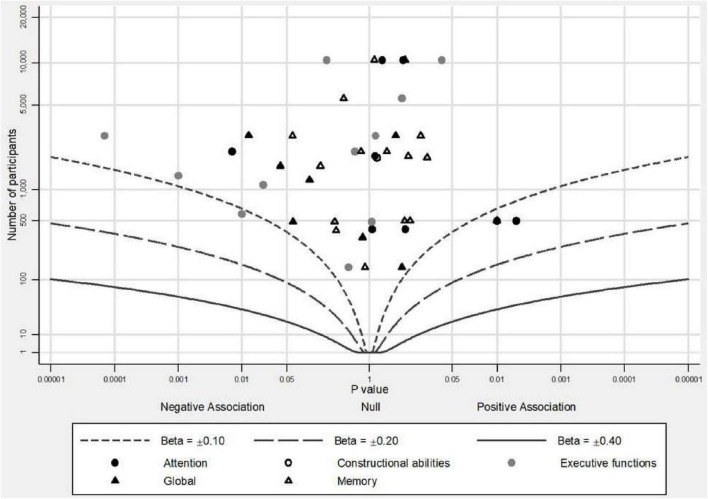
Albatross plot of studies that investigated the association of MetS with continuous outcomes of cognition.

In summary, 7 out of 22 studies presented significant associations of MetS with longitudinal impairment in global cognition and two other studies supported a protective role of MetS on global cognition. Studies were relatively homogeneous with regards to the neuropsychological procedures utilized (MMSE or similar tools) and the criteria for MetS diagnosis (NCEP-ATP III in 15/22). Overall, studies with larger sample sizes tended to report negative associations but studies with smaller sample sizes tended to report positive associations (Figures 2, 3).

#### Memory

Memory, examined in 19 studies, is a complicated cognitive system comprised by many different subsystems. Thus, a great variety of neuropsychological tests is used to assess distinct memory processes, such as verbal or visual memory, depending on the kind of stimuli used in the neuropsychological test. Of the 19 studies, 13 assessed verbal memory (1 in combination with prospective memory task), 3 assessed visual memory and 3 assessed both.

Verbal memory was tested in 11 studies with word-list memory tasks, in 1 study with a short story recall task and in 1 study with both. The recall was requested immediately after stimuli presentation and/or after delay as free recall and/or recognition. Notably, of the 13 studies that examined verbal memory (Komulainen et al., 2007; van den Berg et al., 2007; Yaffe et al., 2007; Knopman et al., 2009; Akbaraly et al., 2010; McEvoy et al., 2012; Watts et al., 2013; Dearborn et al., 2014; Harrison et al., 2015; Gallagher et al., 2016; Shigaeff et al., 2017; Kazlauskaite et al., 2020; Wu and Chen, 2021), only 1 study reported an inverse association between MetS and verbal memory (Komulainen et al., 2007). The rest studies found no association of MetS neither with change in scores (van den Berg et al., 2007; Yaffe et al., 2007; Knopman et al., 2009; McEvoy et al., 2012; Watts et al., 2013; Dearborn et al., 2014; Harrison et al., 2015; Gallagher et al., 2016; Kazlauskaite et al., 2020; Wu and Chen, 2021) nor with cognitive levels (Akbaraly et al., 2010; Watts et al., 2013; Shigaeff et al., 2017; Wu and Chen, 2021) (sample size range 43–6,109 participants, median 1,624 participants, range of follow-up period 1–16 years, median 2 years).

Visual memory was tested in three studies (sample size range 148–7,035 participants, median 1,965 participants, range of follow-up period 1–4 years, median 4 years) with four different neuropsychological procedures. Of the three studies (Raffaitin et al., 2011; Katsumata et al., 2012; Overman et al., 2017), only the largest cohort of 7,035 non-institutionalized elderly (mean age 73.4 years) from three French cities found a consistent association of MetS presence (NCEP-ATP III criteria) with decline in performance (Raffaitin et al., 2011), even after excluding participants that developed dementia during the 4 years of follow-up period.

Three studies assessed memory using neuropsychological tests of both verbal and visual memory, combined as a composite score. Przybycien-Gaweda et al. (2020) and Soldevila-Domenech et al. (2021) used an immediate and delayed recall test with words and abstract designs in 262 Chinese (mean age 65.3 years) and 487 Spaniards (mean age 65.2 years) participants. Both reported no longitudinal association of MetS (NCEP-ATP III and IDF criteria) with decline in memory function after 5 and 3 years, respectively. Bangen et al. (2019) used three subtests from the Wechsler Memory Scale (WMS) (Wechsler, 1945) that included pairs of words, a short story and abstract designs, and reported no association between MetS (NCEP-ATP III criteria) and cognitive performance or cognitive change in a sample of 2,892 Americans (mean age 62.3 years) after 15 years.

In summary, 2 out of 19 studies concluded an association of MetS with prospective decline in memory. Although there was homogeneity in the definition of MetS as most studies (14/19) applied the NCEP-ATP III criteria, there was heterogeneity with regards to the neuropsychological tests used. When cognitive scores were investigated as categorical variables, even if most results were non-significant, there was a tendency for negative point estimates between the presence of MetS and memory function independently of sample size (Figure 2). When cognitive scores were investigated as continuous variables, studies tended to report null associations between MetS and memory independently of sample size (Figure 3).

#### Executive functions

Executive functions are the mental skills necessary for the control of behavior and consist of several cognitive processes (Benson, 2020). Sixteen studies examined executive functions with 17 different neuropsychological tests. The most frequently used neuropsychological test was the Verbal Fluency test that requires from the participants to generate as many words as possible from a given phonemic (Newcombe, 1969) or semantic category (Benton, 1968) in 60 s.

Nine studies evaluated the executive functions using a form of the Verbal fluency test (Knopman et al., 2009; Akbaraly et al., 2010; Raffaitin et al., 2011; Katsumata et al., 2012; McEvoy et al., 2012; Dearborn et al., 2014; Gallagher et al., 2016; Neergaard et al., 2017; Shigaeff et al., 2017). Regarding cognitive levels, one study (Shigaeff et al., 2017) assessed 43 Brazilian elderly at 1 year with 10 different neuropsychological tests and found lower performance of participants with MetS only in the Verbal Fluency test, but two other studies (Akbaraly et al., 2010; Neergaard et al., 2017) with longer follow-up periods (10 and 15 years) and larger sample sizes (4,150 and 1,759 participants) reported no significant differences. It is noteworthy that Akbaraly et al. (2010) found a significant decline in semantic Verbal fluency in fully adjusted models, after controlling for education effects, but this relationship was attenuated in fully adjusted models that accounted for occupation instead of education. The authors suggested that occupation is a more accurate indicator of socioeconomic status, which is linked with cardiovascular health (Schultz et al., 2018), and may mediate the association between MetS and cognition.

Regarding cognitive decline, five studies (Raffaitin et al., 2011; Katsumata et al., 2012; McEvoy et al., 2012; Dearborn et al., 2014; Gallagher et al., 2016) (sample size range 148–7,035 participants, median 5,590 participants, range of follow-up period 3–16 years, median 4 years) reported no association of MetS with rates of cognitive decline but one study (Knopman et al., 2009) that assessed 1,130 individuals (mean age 59 years) with three different neuropsychological tests and after 14 years of follow-up found a significant association of MetS with decline only in the Verbal Fluency test.

The Trail Making Test-Part B (TMT-Part B) (Armitage, 1946) and the Digit Span test-backward condition (Wechsler, 1955) are the next more frequently used neuropsychological tests to examine executive functions. With regard to TMT-Part B, this instrument was used in two studies (McEvoy et al., 2012; Shigaeff et al., 2017). Shigaeff et al. (2017) did not detect any association of MetS with cognitive levels, but McEvoy et al. (2012) recorded a significant rate of decline solely for women. Two studies that used the Digit Span test-backward condition found no statistically significant results neither for cognitive levels (Shigaeff et al., 2017) nor for rates of decline (Kazlauskaite et al., 2020) (sample size range 43–2,149 participants, range of follow-up period 1–13 years).

Other neuropsychological tests widely used to assess executive functions are the Clock Drawing test (Sunderland et al., 1989), the Wisconsin Card Sorting Test (WCST) (Grant and Berg, 1948) and the Stroop Test (Stroop, 1935). Viscogliosi et al. (2016) investigated the relation of the presence of MetS, defined by NCEP-ATP III criteria, with executive functions using the Clock Drawing test and reported a greater one-year decline for participants with MetS than those without. Shigaeff et al. (2017) did not find any difference between MetS group and a group of controls in performance on WCST test that demands ability to rapidly change strategies. However, van den Berg et al. (2007) reported decelerated decline at the five-year follow-up period in participants with MetS older than 85 years old using the Stroop test, a test created to assess the ability to inhibit cognitive interference. Authors attributed this result to survival bias.

Akbaraly et al. (2010) and Creavin et al. (2012) measured executive functions with the Alice Heim 4 test-part 1 (AH4) (Heim, 1970), a test of fluid intelligence comprising items of mathematical and verbal reasoning. Creavin et al. (2012) examined 1,225 men (median age 52.8 years) from United Kingdom and found that men with MetS defined by WHO criteria had lower scores on AH4 after 13 years. On the other hand, Akbaraly et al. (2010) found no statistical difference in the performance of participants with and without MetS defined by NCEP-ATP III criteria, in a larger cohort of 4,150 Spanish participants (mean age 61.1 years) using the same test.

Lastly, combined scores with some of the above mentioned neuropsychological tests were used by three research teams but none of them concluded an association of MetS on decline in this cognitive domain (Bangen et al., 2019; Przybycien-Gaweda et al., 2020; Soldevila-Domenech et al., 2021). They combined the score of 5 or 6 different neuropsychological tests and examined 2,892, 262 and 482 participants for 15, 5, and 3 years, respectively. Bangen et al. (2019) did find lower levels of cognitive performance at follow-up, but participants with MetS had also lower levels of cognitive performance at baseline.

In summary, 5 out of 16 studies showed evidence of negative impact of MetS on executive functions longitudinally and 1 study showed evidence of protective role of MetS. NCEP-ATP III criteria were the most commonly used for the definition of MetS (10/16) but a similar form of test was used only in 9 out of 16 studies. Thus, heterogeneity of the neuropsychological procedures used could have an important role for the final result reported. In Figure 2, where cognitive scores were analyzed as categorical variables, there were two insignificant negative results, but in Figure 3 presenting cognitive scores analyzed as continuous variables, studies with smaller sample sizes leaned to the left side of the plot indicating negative associations of MetS with executive functions, whereas studies with larger sample sizes leaned to the right side of the plot indicating positive point estimates, but all had insignificant results.

#### Attention

Attention is a combination of mechanisms that enable a person to select, sustain and modulate focus on relevant stimuli to determine the behavior while ignoring other simultaneous stimuli (Chun et al., 2011). Within the studies reviewed, 12 studies examined the cognitive domain of attention. Five studies investigated the association of MetS with impairment in attention using a version of the Digit Symbol Substitution test (DSST) (Wechsler, 1981). This procedure consists of pairs of digits and symbols and participants are asked to match as fast as possible the digits with symbols. Two studies presumed an association of MetS with rates of change in attention (van den Berg et al., 2007; Kazlauskaite et al., 2020), whereas the rest three studies, two of which used sample from the same cohort, did not report any significant association (Knopman et al., 2009; Dearborn et al., 2014; Overman et al., 2017). Kazlauskaite et al. (2020) examined 2,149 American women (mean age 50.7 years) and 10-year decline in attention was consistently associated with the presence of MetS defined by Cardiometabolic Health Alliance criteria, even after controlling for sociodemographic factors, lifestyle factors, other medical conditions and practice effects. On the contrary, van den Berg et al. (2007) found a slower decline rate in attention in participants with MetS defined by NCEP-ATP III criteria. However, participants included in this study were participants from the Leiden 85-plus Study, a prospective population-based study of individuals older than 85 years old.

Three additional studies used a version of DSST test combined with other tests to create a composite factor score of attention. None of them concluded any association of MetS with performance level (Komulainen et al., 2007; Watts et al., 2013) or decline (Watts et al., 2013; Przybycien-Gaweda et al., 2020) in attention. However, all studies had small sample sizes (101, 73, and 262 participants, respectively).

Shigaeff et al. (2017) used the Trail Making Test-Part A and the Digit Span test-forward condition to assess attention of 43 individuals matched for age group and educational level (23 MetS, 20 controls). At one-year follow-up, no between groups difference on cognitive performance was noted.

Attention was further tested with reaction time tests and again outcomes were similar in participants with and without a MetS diagnosis, defined by WHO (Creavin et al., 2012) or NCEP-ATP III criteria (Harrison et al., 2015). Creavin et al. (2012) measured attention with a four-choice reaction time task (Nissen and Bullemer, 1987) in 1,225 men from United Kingdom aged 45-59 years for 13 years and did not detect any association of performance with MetS diagnosis. Harrison et al. (2015) investigated the effect of MetS on attention using combined scores from three different reaction time tasks from the Cognitive Drug Research battery (Simpson et al., 1991). She and her colleagues examined 845 community-dwelling and institutionalized adults over 85 years old for up to 5 years and reported no association of MetS with cognitive changer over time, even after excluding participants with cognitive impairment at baseline.

Wu and Chen (2021) examined 5,693 Taiwanese individuals (mean age 63.67 years) using the MMSE during a single 10-year follow-up but reported the hazard ratio for each domain separately. Orientation, registration and concentration are the domains of the MMSE that are included in the broad cognitive ability of attention. Of these three, only the orientation had significantly lower scores in all models (unadjusted and fully adjusted) and higher cumulative risk of decline in participants with MetS.

In summary, 2 out of 12 studies established an association of MetS with prospective decline in attention and 1 out of 12 studies established a slower decline rate in attention in participants with MetS. The most commonly used criteria for the definition of MetS were NCEP-ATP III (8/12) but there were 11 different neuropsychological tests used. In Figure 2, there was no clear tendency except for one study that presented a significant negative estimate, meaning that the presence of MetS was associated with decline in attention. In Figure 3, there was an inclination for positive point estimates (although not significant) between MetS and attentional abilities apart from one study that presented a significant negative estimate.

#### Visuoconstructive abilities

Three studies examined MetS diagnosis in relation to visuoconstructive abilities, namely the abilities to analyze the elements and the spatial relationships of a visual stimulus and to reproduce this stimulus (Benton, 1967). Shigaeff et al. (2017) requested from 43 participants older than 65 years with and without MetS to reproduce two-dimensional, geometric patterns using two-color cubes and found no difference between their performance at 1 year follow-up period, whereas Wu and Chen (2021) using the copying task of intersecting pentagons from the MMSE predicted lower performance and higher risk of decline in visuoconstructive abilities in participants with MetS after 10 years in a cohort of 5,693 participants (mean age 63.67 years).

Overman et al. (2017) requested from 1,913 men aged 40–79 years to copy an abstract figure as accurately as possible and reported no association of MetS with the performance on this task neither using the continuous score of change nor using the categorical variable of cognitive change (decline vs. no change vs. improvement). However, when the categorical variable of cognitive change was analyzed separately for middle-aged participants (<65 years) and older participants (≥65 years), there was a significant association between a MetS diagnosis and impairment on this visuoconstructional task, but only for the group of older participants.

In summary, 1 out of 3 studies reported higher risk of decline in visuoconstructive abilities in participants with MetS. All studies used the NCEP-ATP III criteria for the definition of MetS, but different neuropsychological procedures to assess visuoconstructive abilities.

#### Language

The longitudinal association of MetS with language was assessed in three studies. Shigaeff et al. (2017) used the Boston Naming Test (Kaplan et al., 2001), a confrontation naming test with 60 black and white line drawings of objects, did not find differences in the performance of 43 participants with and without MetS, defined by NCEP-ATP III criteria. Wu and Chen (2021) using the language tasks of the MMSE (confrontation naming, repetition, sentence production and understanding) did not find difference in the performance of 5,693 participants with and without MetS in the fully adjusted models, but did find higher risk of decline for participants with MetS in 10 years. Akbaraly et al. (2010) using the Mill Hill Vocabulary test (Deltour, 1993), a list of 33 stimulus words with six multiple-choice synonyms, reported lower performance for participants with MetS defined by NCEP-ATP III criteria after 10 years in a cohort of 4,150 participants (mean age 61.1 years).

In conclusion, two studies out of three indicated a significant relationship between the diagnosis of MetS and decline in language abilities.

### Bibliometric analysis

Supplementary Figure 1 depicts the constant interest in investigating the relationship of MetS with cognitive decline as almost every year since 2004 there was one longitudinal study published in this field, reaching the highest peak in 2017 when four studies were published.

Supplementary Figure 2 shows the trends in the cognitive domains examined. The four basic domains that are repeatedly investigated over the years are global cognition, executive functions, memory and attention. The domains of language and visuoconstructive abilities are investigated less frequently and in the more recent years. It is possible that these cognitive processes are considered less vulnerable to the consequences of MetS since they are located in specific brain regions (Geschwind, 1972; Goodale and Milner, 1992), not known to be affected by the subcortical lesions associated with the presence of MetS (Alfaro et al., 2018).

Research teams from USA contributed 10 out of 30 included studies, followed by research teams from China that contributed three studies. United Kingdom contributed two studies as single country publications and two studies in collaboration with research teams from other countries (Supplementary Figure 3). This might be explained by the fact that the USA and China are the leading countries in investment of funds on research according to the UNESCO Institute for Statistics (UNESCO, 2017).

The most cited study is the first longitudinal study published of Yaffe et al. (2004). The next most cited studies are the subsequent published study of Yaffe et al. (2007) and a study of Knopman et al. (2009) from the ARIC cohort, a large prospective epidemiologic study in USA that began in 1987 (Supplementary Table 2).

The most common keywords reported by the authors and keywords-plus reported by SCOPUS database were the basic terms regarding the exposure “metabolic syndrome” and “metabolic syndrome X,” the outcome “cognition,” “cognitive decline” and “cognitive defect,” and the keywords describing the sample “aging,” “elderly,” “aged,” “male” and “female” (Supplementary Table 3). The figure of trends in topics reveals that these terms are present throughout the years (Supplementary Figure 4).

Interestingly, in the first years the term “mini mental examination state examination” is present implying its wide use to examine cognition, whereas in the more recent years there is no presence of any neuropsychological test except for the general term “memory,” implying the use of more diverse neuropsychological tests that do not reach the threshold in order to be depicted on the figure (Supplementary Figure 4).

## Discussion

The purpose of the present systematic review was to summarize the results from studies that examined the longitudinal effect of MetS on cognition overall and on the distinct cognitive domains. Prior evidence is mixed. Two previous meta-analyses supported an association of MetS with cognitive decline (Siervo et al., 2014; González-Castañeda et al., 2021). Nevertheless, both mixed different neuropsychological tests and one reported only a weak association (Siervo et al., 2014). Previous systematic reviews generally indicated a relationship between the presence of MetS and poor cognition but none took into consideration separate cognitive domains (Hao et al., 2011; Crichton et al., 2012; Yates et al., 2012; Farruggia and Small, 2019; Tahmi et al., 2021). Finally, the results of the present study are compatible with two systematic reviews that reached inconclusive results (Assuncao et al., 2018; Alcorn et al., 2019).

Thirty studies were identified with seventeen concluding that the presence of MetS accelerates cognitive decline, whereas two showing that the presence of MetS decelerates cognitive decline. In contrast, for domain-specific results, the majority of the literature showed non-significant results. The null domain-specific results could be attributed to a more diffused pattern of brain pathology in white matter microstructure integrity and gray matter volume linked with the presence of MetS (Alfaro et al., 2018; Kotkowski et al., 2019), and not with specific focal alterations which would lead to discrete impairments to certain cognitive domains. Therefore, an overall pattern of decline in cognition can be anticipated in individuals with MetS but may not be evident in specific cognitive abilities.

Another potential explanation may be important effect modifiers that could determine if a person with MetS will eventually develop cognitive decline or not. For example, two American studies (Yaffe et al., 2004, 2007) found that inflammation markers (such as C-reactive protein) moderated the association across people with MetS, with high levels of inflammation markers indicating significantly higher rates of decline in global cognitive tests. Inflammation may have a direct negative impact on cognition (Sartori et al., 2012) or a synergistic effect with MetS. Similarly, Bangen et al. (2019) found that among people with MetS, non-carriers of apolipoprotein (APOE) ε4 in contrast with carriers, had lower level of cognitive functioning in the executive function domain. This finding was attributed to the adverse effects that insulin resistance exerts on non-carriers of APOE ε4 to a greater extent than on carriers (Avgerinos et al., 2018). However, it is important to note that both studies had small sample sizes to adequately study interactions.

Moreover, the exact constellation of components that constitute the MetS diagnosis for each participant could cause discrepant outcomes. It is probable that an individual with obesity, hypertension and low HDL levels may have a different cognitive profile from an individual with diabetes, hypertriglyceridemia and hypertension. For instance, McEvoy et al. (2012) found that MetS was associated with decline in TMT-Part B in women with diabetes but not in those without.

Many methodological issues could also contribute to these disparate results. Exposure misclassification due to heterogeneity in the specific components and their cutoffs used for the definitions of MetS may explain the inconsistency of the studies’ findings and poses a major threat to the replicability of the results. In addition, the plethora of cognitive tests used, even in the assessment of the same cognitive domain, have different sensitivity that could have affected the ability to detect minor cognitive deficits. Lastly, large variations across the included studies, such as sample size, distribution of baseline characteristics (e.g., age, education, gender), follow-up duration and the covariates accounted for, could partly explain the inconsistent findings. These methodological issues precluded the conduct of meta-analysis and obscures a cohesive summary of this literature.

The results from the bibliometric analysis indicated a growing interest in the domain of cognitive function in people with MetS. Until today, most studies are conducted by research teams in USA and China; similar studies in other countries are needed. Moreover, most studies examined the domains of global cognition, executive functions, memory and attention but cognitive tasks that reflect the remaining cognitive domains (ex. visuoconstructive abilities, language) or real-life situations should be addressed in future studies. Overall, this systematic review found limited evidence of an association between the presence of MetS and concomitant prospective decrements in cognitive abilities of a particular domain. The existing literature demonstrates the need for the development of a global consensus regarding the definition of MetS and for standardized procedures of cognitive examination in order to improve comparability among studies. Future studies following more standardized methodologies, such as the utilization of similar validated neuropsychological tests, follow-up periods and statistical analysis methods, are required to elucidate the impact of MetS on cognition and its underlying mechanisms.

## Data availability statement

The data presented in this Systematic Review is included in the article/Supplementary material, further inquiries can be directed to the corresponding author/s.

## Author contributions

KT and MK: conceptualization of review. MK: literature search and original draft. KT, GM, and MK: critical revisions including article exclusion and inclusion and risk of bias assessment. EB and MK: bibliometric analysis. KT, EA, GM, and EB: review and editing. All authors read and approved the final manuscript.
