# Supplementary figures and images for: Metabolic syndrome and cognition: A systematic review across cognitive domains and a bibliometric analysis
Source: Front Psychol. 2022 Nov 9;13:981379. doi: 10.3389/fpsyg.2022.981379 (PMC9682181; doi:10.3389/fpsyg.2022.981379)

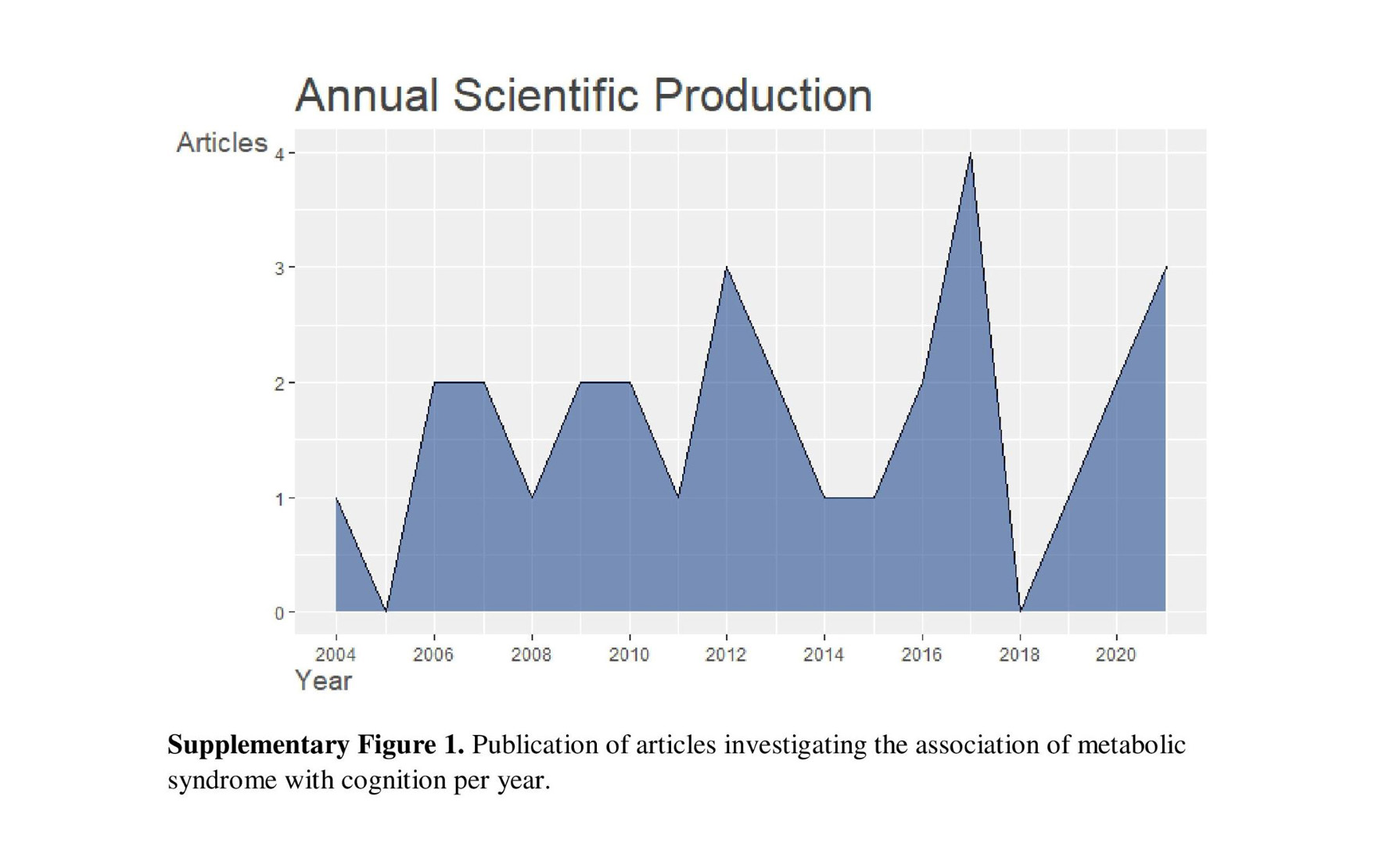

Supplement: Supplementary file 1 [file Image_1.JPEG]

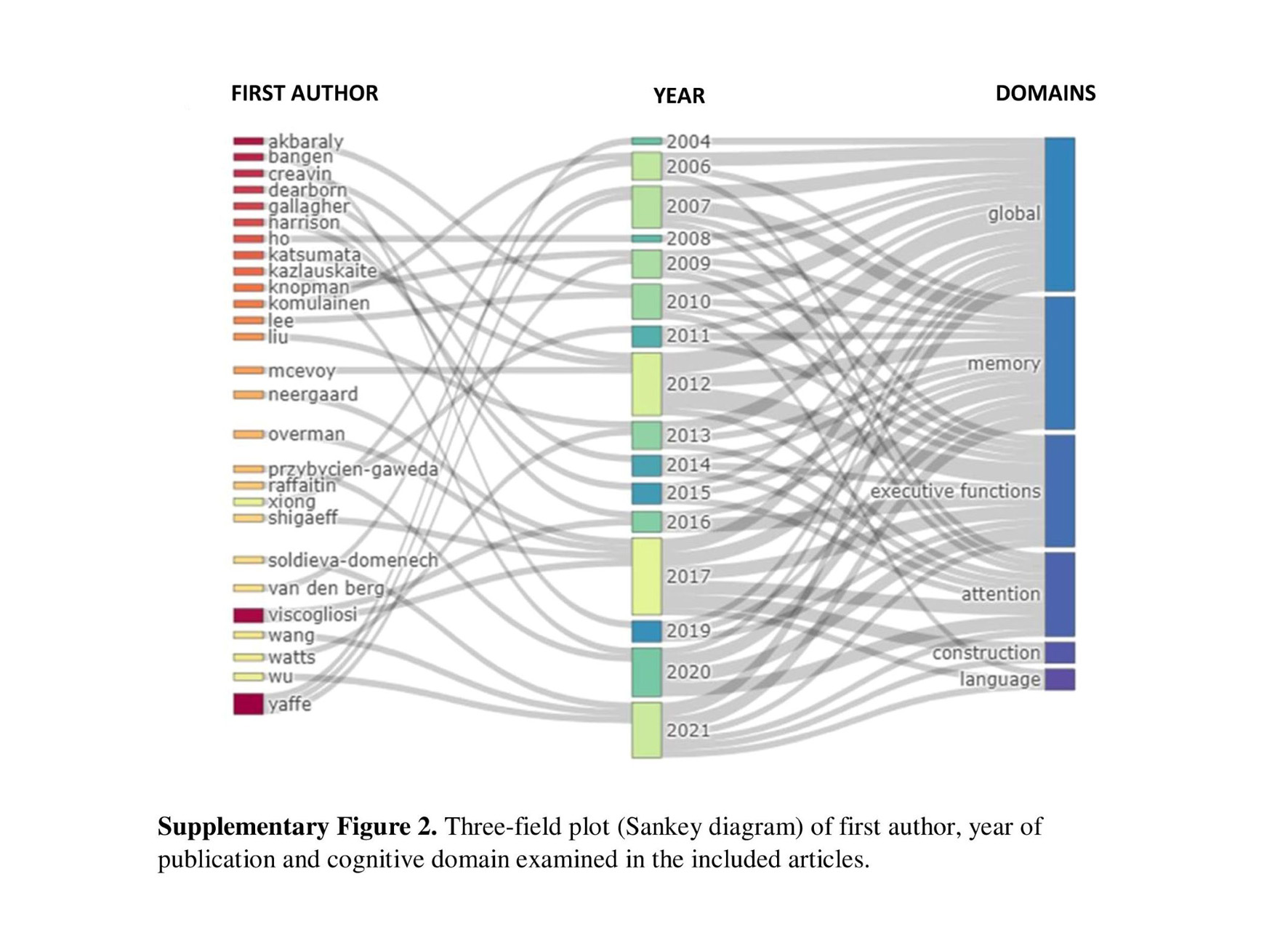

Supplement: Supplementary file 2 [file Image_2.JPEG]

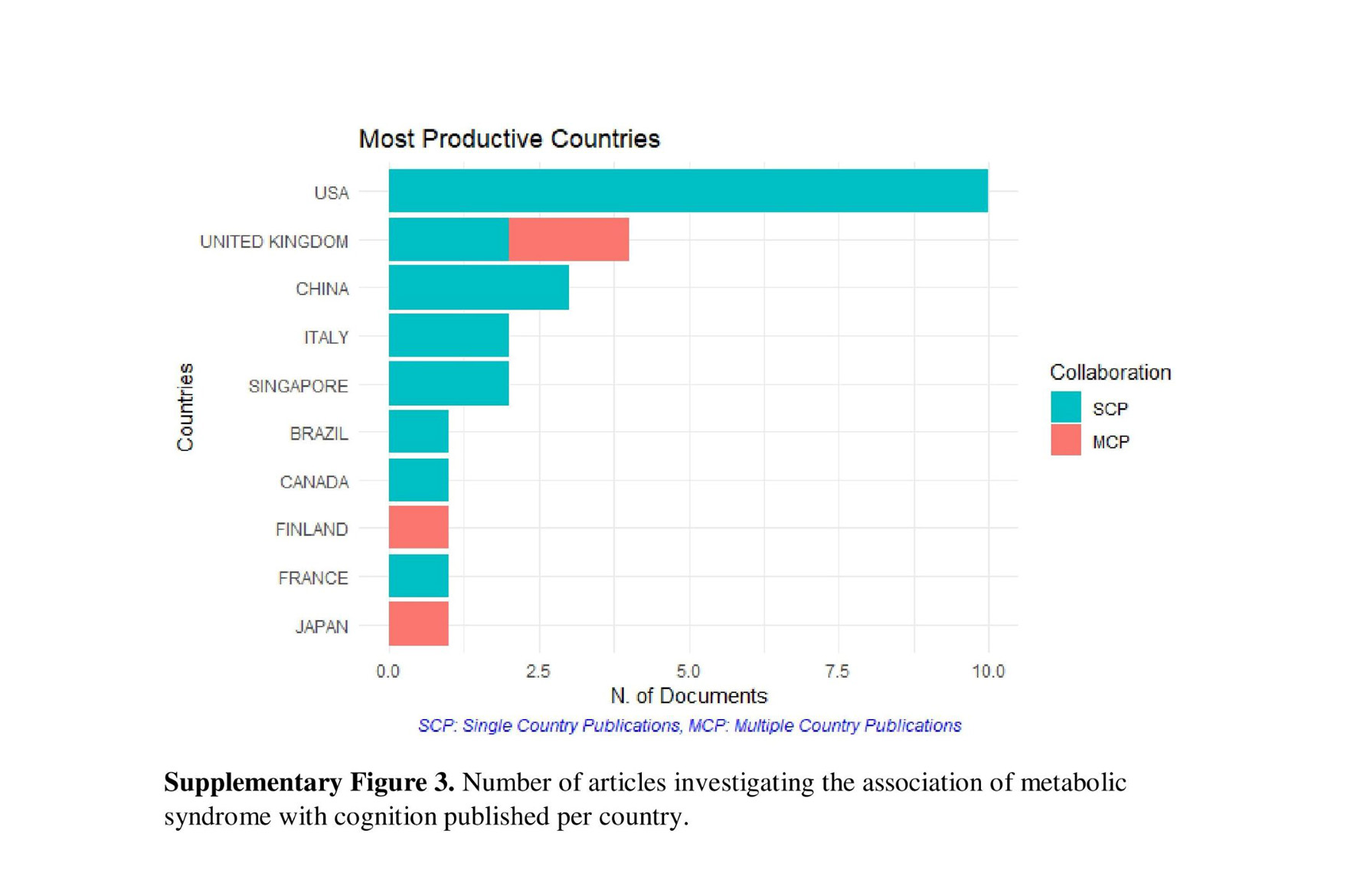

Supplement: Supplementary file 3 [file Image_3.JPEG]

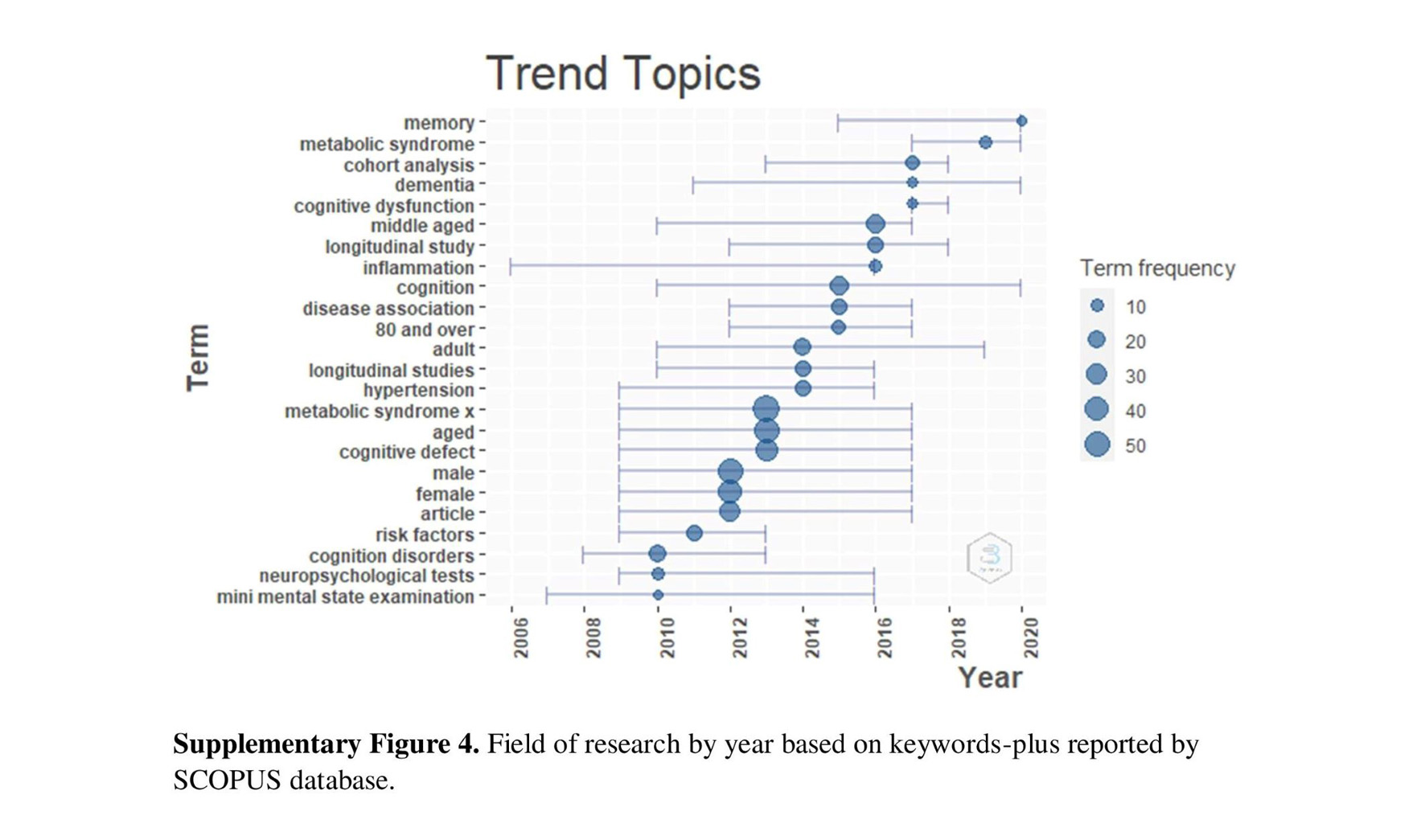

Supplement: Supplementary file 4 [file Image_4.JPEG]
