# Supplementary material for: Metabolic syndrome and cognition: A systematic review across cognitive domains and a bibliometric analysis
Source: Front Psychol. 2022 Nov 9;13:981379. doi: 10.3389/fpsyg.2022.981379 (PMC9682181; doi:10.3389/fpsyg.2022.981379)
Supplement: Supplementary file 5 [file Table_1.DOCX]

Supplementary Material

**Supplementary Table 1.** Quality assessment of the included studies according to Newcastle-Ottawa Scale for cohort studies.

| **LEAD AUTHOR (YEAR)** | **SELECTION** | | | | **COMPARAMBILITY** | **OUTCOME** | | | **TOTAL** |
| --- | --- | --- | --- | --- | --- | --- | --- | --- | --- |
|  | Representativeness of the exposed cohort (generalizability) | Selection of the non exposed cohort (selection bias) | Ascertainment of exposure (exposure measurement error) | Demonstration that outcome of interest was not present at start of study (reverse causation bias) | Comparability of cohorts on the basis of the design or analysis (residual confounding) | Assessment of outcome (outcome measurement error) | Was follow-up long enough for outcomes to occur (adequacy of follow-up) | Adequacy of follow up of cohorts (bias due to loss to follow-up) |  |
| Akbaraly (2010) | 0 | 1 | 1 | 0 | 2 | 1 | 1 | 0 | 6 |
| Bangen (2019) | 1 | 1 | 1 | 1 | 1 | 1 | 1 | 1 | 8 |
| Creavin (2012) | 1 | 1 | 1 | 0 | 1 | 0 | 1 | 0 | 5 |
| Dearborn (2014) | 1 | 1 | 1 | 0 | 2 | 1 | 1 | 1 | 8 |
| Gallagher (2016) | 1 | 1 | 1 | 1 | 0 | 1 | 1 | 0 | 6 |
| Harrison (2015) | 1 | 1 | 1 | 0 | 2 | 1 | 1 | 0 | 7 |
| Ho (2008) | 1 | 1 | 1 | 1 | 2 | 1 | 0 | 0 | 7 |
| Katsumata (2012) | 1 | 1 | 1 | 1 | 1 | 1 | 0 | 0 | 6 |
| Kalzauskaite (2020) | 1 | 1 | 1 | 0 | 2 | 1 | 1 | 1 | 8 |
| Knopman (2009) | 1 | 1 | 1 | 0 | 1 | 1 | 1 | 0 | 6 |
| Komulainen (2007) | 1 | 1 | 1 | 1 | 1 | 1 | 1 | 0 | 7 |
| Lee (2010) | 0 | 1 | 1 | 0 | 1 | 1 | 0 | 0 | 4 |
| Liu (2013) | 0 | 1 | 1 | 1 | 0 | 1 | 0 | 1 | 5 |
| McEvoy (2012) | 1 | 1 | 1 | 0 | 1 | 1 | 1 | 0 | 6 |
| Neergaard (2017) | 1 | 1 | 1 | 1 | 1 | 1 | 1 | 1 | 8 |
| Overman (2017) | 1 | 1 | 1 | 0 | 2 | 1 | 1 | 0 | 7 |
| Przybycien-Gaweda (2020) | 1 | 1 | 1 | 1 | 2 | 1 | 1 | 0 | 8 |
| Raffaitin (2011) | 1 | 1 | 1 | 1 | 2 | 1 | 1 | 0 | 8 |
| Shigaeff (2017) | 1 | 1 | 1 | 1 | 0 | 1 | 0 | 1 | 6 |
| Soldieva-Domenech (2021) | 1 | 1 | 0 | 1 | 2 | 1 | 0 | 1 | 7 |
| van den Berg (2007) | 1 | 1 | 1 | 0 | 1 | 1 | 1 | 1 | 7 |
| Viscogliosi (2016) | 0 | 1 | 1 | 1 | 0 | 1 | 0 | 1 | 5 |
| Viscogliosi (2017) | 1 | 1 | 1 | 0 | 1 | 1 | 1 | 0 | 6 |
| Wang (2021) | 1 | 1 | 1 | 1 | 1 | 1 | 1 | 1 | 8 |
| Watts (2013) | 1 | 1 | 1 | 1 | 1 | 1 | 0 | 1 | 7 |
| Wu (2021) | 1 | 1 | 1 | 0 | 2 | 1 | 1 | 0 | 7 |
| Xiong (2006) | 0 | 0 | 1 | 1 | 1 | 0 | 1 | 1 | 5 |
| Yaffe (2004) | 1 | 1 | 1 | 1 | 0 | 1 | 1 | 1 | 7 |
| Yaffe (2007) | 1 | 1 | 1 | 0 | 2 | 1 | 0 | 1 | 7 |
| Yaffe (2009) | 1 | 1 | 1 | 1 | 1 | 1 | 1 | 1 | 8 |
