# Supplementary material for: Metabolic syndrome and cognition: A systematic review across cognitive domains and a bibliometric analysis
Source: Front Psychol. 2022 Nov 9;13:981379. doi: 10.3389/fpsyg.2022.981379 (PMC9682181; doi:10.3389/fpsyg.2022.981379)
Supplement: Supplementary file 6 [file Table_2.DOCX]

Supplementary Material

**Supplementary Table 2.** Top cited articles investigating the association of metabolic syndrome with cognition.

| First author | Year of publication | Total citations |
| --- | --- | --- |
| Yaffe | 2004 | 760 |
| Yaffe | 2007 | 171 |
| Knopman | 2009 | 162 |
| van den Berg | 2007 | 158 |
| Yaffe | 2009 | 156 |
