# Supplementary material for: Metabolic syndrome and cognition: A systematic review across cognitive domains and a bibliometric analysis
Source: Front Psychol. 2022 Nov 9;13:981379. doi: 10.3389/fpsyg.2022.981379 (PMC9682181; doi:10.3389/fpsyg.2022.981379)
Supplement: Supplementary file 7 [file Table_3.DOCX]

Supplementary Material

**Supplementary Table 3.** Most common reported keywords by authors and by SCOPUS database.

| AUTHORS KEYWORDS | | KEYWORDS-PLUS | |
| --- | --- | --- | --- |
| Keyword | Frequency | Keyword | Frequency |
| Metabolic Syndrome | 17 | Metabolic Syndrome X | 54 |
| Cognition | 9 | Aged | 50 |
| Cognitive Decline | 6 | Male | 42 |
| Aging | 4 | Female | 40 |
| Elderly | 4 | Cognive Defect | 32 |
